# VennPainter: A Tool for the Comparison and Identification of Candidate Genes Based on Venn Diagrams

**DOI:** 10.1371/journal.pone.0154315

**Published:** 2016-04-27

**Authors:** Guoliang Lin, Jing Chai, Shuo Yuan, Chao Mai, Li Cai, Robert W. Murphy, Wei Zhou, Jing Luo

**Affiliations:** 1 Key Laboratory for Animal Genetic Diversity and Evolution of High Education in Yunnan Province, School of Life Sciences, Yunnan University, Kunming, 650091, China; 2 School of Software, Yunnan University, Kunming, 650091, Yunnan, China; 3 State Key Laboratory for Conservation and Utilization of Bio-resource, Yunnan University, Kunming, 650091, Yunnan, China; 4 State Key Laboratory of Genetic Resources and Evolution, Kunming Institute of Zoology, The Chinese Academy of Sciences, Kunming, 650223, Yunnan, China; 5 Kunming College of Life Science, University of Chinese Academy of Sciences, Kunming, 650000, China; 6 Centre for Biodiversity and Conservation Biology, Royal Ontario Museum, Toronto, M5S 2C6, Canada; 7 School of Computer and Science, Fudan University, Shanghai, 200433, China; University of Toronto, CANADA

## Abstract

VennPainter is a program for depicting unique and shared sets of genes lists and generating Venn diagrams, by using the Qt C++ framework. The software produces Classic Venn, Edwards’ Venn and Nested Venn diagrams and allows for eight sets in a graph mode and 31 sets in data processing mode only. In comparison, previous programs produce Classic Venn and Edwards’ Venn diagrams and allow for a maximum of six sets. The software incorporates user-friendly features and works in Windows, Linux and Mac OS. Its graphical interface does not require a user to have programing skills. Users can modify diagram content for up to eight datasets because of the Scalable Vector Graphics output. VennPainter can provide output results in vertical, horizontal and matrix formats, which facilitates sharing datasets as required for further identification of candidate genes. Users can obtain gene lists from shared sets by clicking the numbers on the diagram. Thus, VennPainter is an easy-to-use, highly efficient, cross-platform and powerful program that provides a more comprehensive tool for identifying candidate genes and visualizing the relationships among genes or gene families in comparative analysis.

## Introduction

In comparative genomics, the visualization of results can help viewers discover correlations and trends in large datasets [1–4]. Many methods can visualize statistical analysis (e.g., scatter diagrams, line graphs, and histograms) [2,3,5–8], biological networks (e.g., pathway and functional networks) [7,9–11] and comparisons of large-scale ‘omic’ data (e.g., clusters, heatmaps, and circsters) [1,3,4,6,12]. Venn diagrams, first developed by John Venn in 1880 [13], are widely used for comparing multiple genomic, transcriptomic and proteomic datasets due to their ease-of-interpretation and graphical simplicity [14–21]. These diagrams help to identify candidate genes and gene networks for downstream analyses. For example, the simple n-Venn diagram is a collection of n simple intersecting closed curves in the plane. It indicates the relationships among datasets, including intersections, sums, complements [13,22]. The curves divide the plane into 2^n^-1 distinct intersections, each defined by its intersection of the interior or exterior of each of the curves [23]. Generally, the Classic Venn diagram deciphers no more than four sets. The development of Symbolic Logic has facilitated several approaches for constructing Venn diagram with more than five sets, including Classic, Edwards’, Lewis Carroll’s and Nested Venn diagrams [24,25]. Edwards’ and Nested methods might generate Venn diagram for an infinite number sets, but the partition of sets among multiple datasets might have complex associations because distinct open regions increase exponentially with the increase in set-number. This makes it difficult to generate intuitive diagrams that display associations among datasets.

Many open access programs can generate Venn diagrams, such as Venny [26], VennDiagram [27], BioVenn [28], GeneVenn [29], 4-way Venn Diagram Generator, DrawVenn, VennMaster [30], VennPlex [31], VennTure [32] and others. However, these programs have some limitations. For example, DrawVenn requires the manual drawing of diagrams and it cannot process data. VennMaster [30] provides area-proportional Euler diagrams for functional GO analysis of microarrays only. VennPlex [31] compares and visualizes datasets with differentially regulated data points. Powerful VennDiagram [27] generates Venn and Euler diagrams in R and it provides a large number of customizable features. Unfortunately, its command-line operation is not user-friendly. VennTure [32] can generate six-sets Venn diagrams with a graphic user interface (GUI), yet it consumes large amounts of memory and has low computational efficiency. Venny [26], BioVenn [28], GeneVenn [29], and 4-way Venn Diagram Generator are web applications. Despite their power and being user-friendly, none of them can evaluate more than four datasets. The latest program, jVenn [33], can handle six input lists at most but only provides Classic and Edwards’ Venn diagrams.

**Fig 1:**
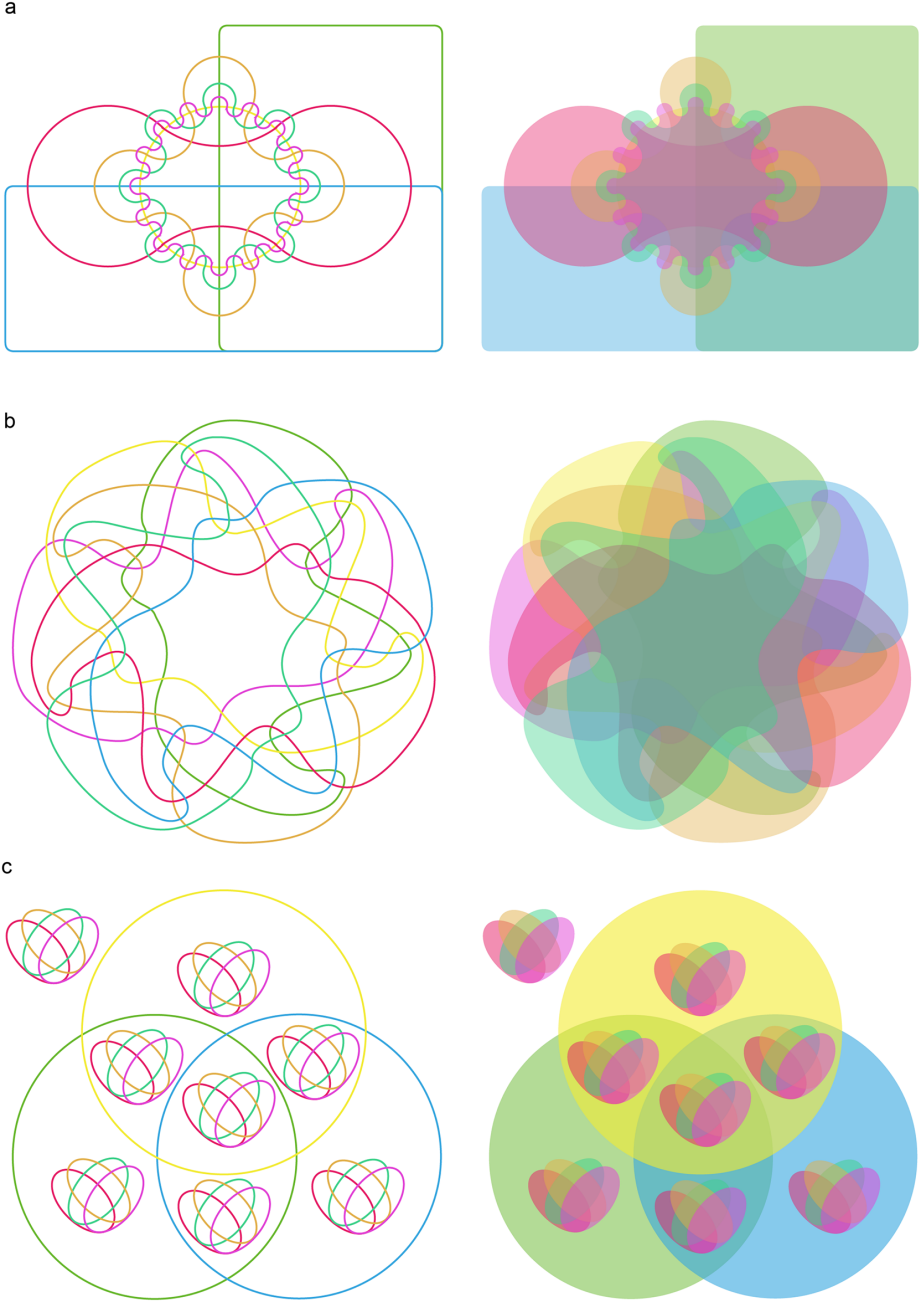
Venn diagrams for seven sets. **(a)** Edward’s Venn diagram constructed with cogwheels, which become smaller with increasing numbers of sets. With seven sets, this made it hard to fill intersections with a number. **(b)** Venn diagram constructed with irregular curves. Some intersections are unclear. **(c)** Nested Venn diagram places its intersections more evenly and regularly, which facilitates accurate interpretation.

Available programs generate no more than six-set Venn diagrams and only support Classic and Edwards’ Venn layouts. Larger datasets often present an insurmountable challenge to deciphering and drawing Venn diagrams of shared relationships manually. This complexity might explain the dearth of applications [34–37]. To rectify this limitation and address Venn-based demands, we report the development of VennPainter, a program that introduces a new nested Venn layout. Fig 1 illustrates seven-set Edwards’ (Fig 1a) and Classic’s (Fig 1b) Venn diagrams. The former illustrates that intersections become smaller with increasing numbers of sets, which presents a challenge for interpretation. The irregular curves of the latter approach are equally challenging. In comparison, the nested Venn (Fig 1c) is far more easily interpreted. VennPainter incorporates the nested Venn layout and increases the number of allowable datasets up to eight with diagram output. It also offers text output of up to 31 datasets for downstream analyses. Further, VennPainter elevates computational efficiency.

## Implementation and Method

### VennPainter and its availability

**Fig 2:**
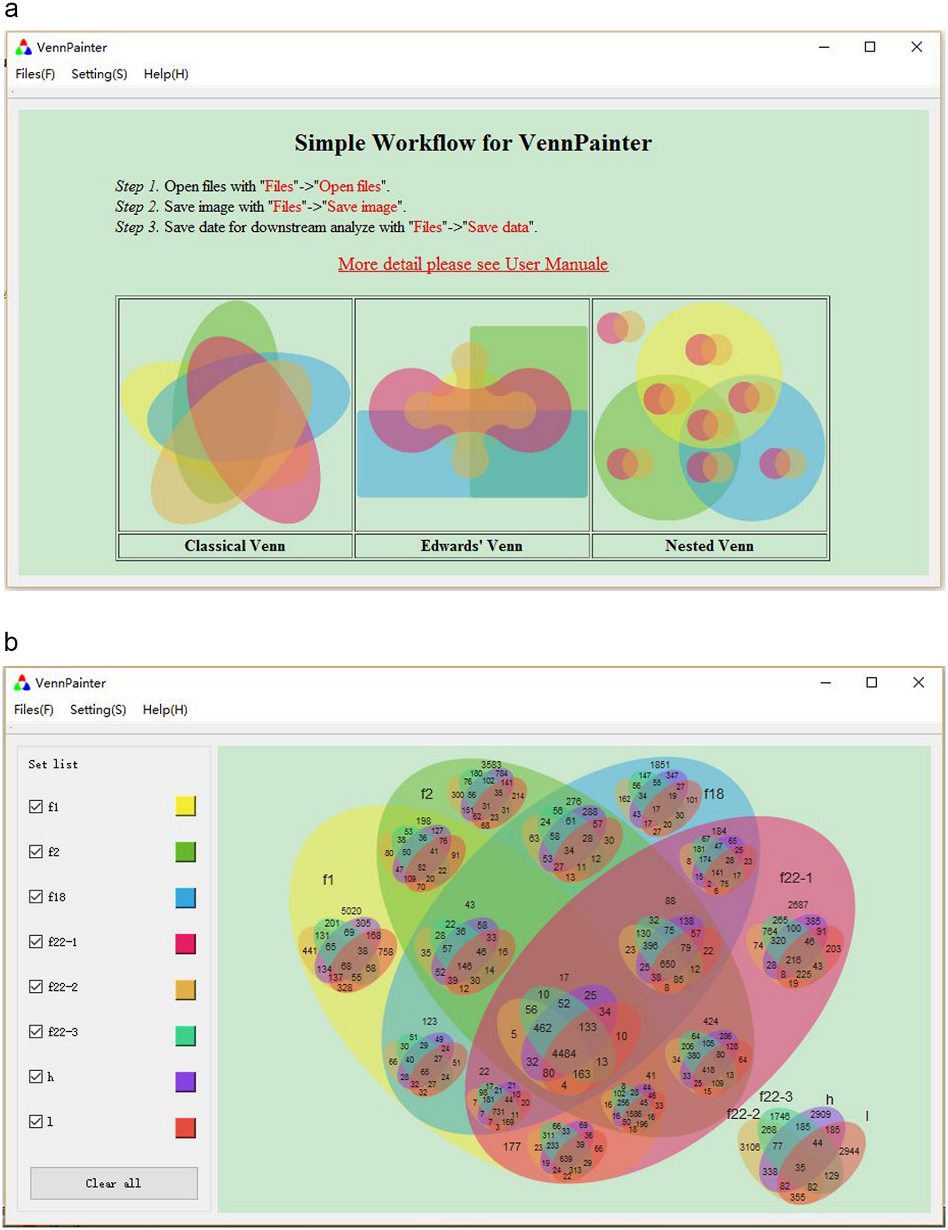
VennPainter GUI. The simple graphical user interface (GUI) for VennPainter. **(a)** A simple workflow appears on the canvas after loading VennPainter. **(b)** After loading data, the GUI control panel will appear for customizing colors.

VennPainter (Fig 2) was developed with Qt 4.8.5 under its LGPL v2.1 license. The Qt C++ framework was chosen for its cross-platform capabilities, open-source nature, and secure language construction for communicating between objects (signals and slots) (http://qt-project.org/). For data sets ranging from nine to 31, VennPainter provides vertical, horizontal and matrix text-based formats for the benefit of downstream analyses. The user manual and basic instructions appear in the initial interface of the program, and can be downloaded together with VennPainter at https://github.com/linguoliang/VennPainter/.

### Algorithm

VennPainter uses set-theory to generate Venn diagrams. The intersection is defined as follow:

$$A\cap B\;=\;\left\{x:x\in A\wedge x\in B\right\}$$

and its complement:

$$B\setminus A\;=\;B\cap A^{∁}\;=\;\left\{x:x\in B\wedge x\notin A\right\}$$

Technically, integer *a*_*x*_ is assigned to label element *x*.

$$\forall x\in \overset{n}{\underset{i\;=\;1}{⋃}}A_{i},\;1\leq n\leq 31$$

*a*_*x*_ can represent the following:

$$a_{x}\;=\;\sum_{i\;=\;1}^{n}b_{i},\;1\leq n\leq 31$$

and

$$b_{i}=\{\begin{array}{rr} 2^{i-1}, & x\in A_{i}\\ 0, & x\notin A_{i} \end{array}$$

**Fig 3:**
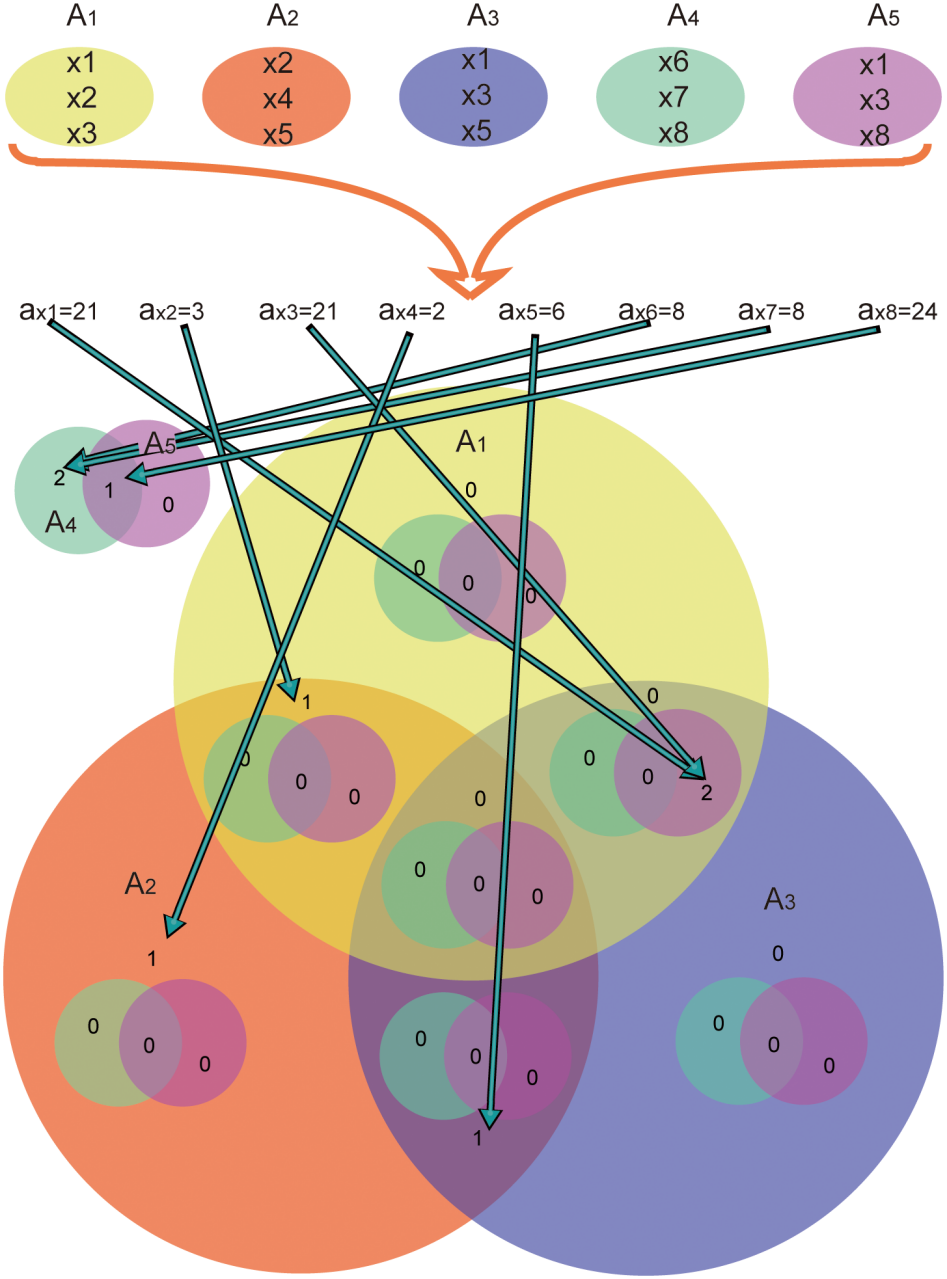
Work flow of VennPainter. The workflow has three steps: 1, calculate all label elements; 2, find the corresponding label in the Venn diagram; and 3, count all intersections and draw Venn diagrams.

Thus, if *a*_*x*1_ = *a*_*x*2_, then *x*1 and *x*2 belong to the same intersection. VennPainter labels every intersection ***U***_***m***_ with an integer $c_{U_{m}}$ in the Venn diagram (S1, S2 and S3 Figs). If $a_{x}=c_{U_{m}}$, then ***x*** ∈ ***U***_***m***_. The flowchart (Fig 3) shows how VennPainter works.

### Adapted Venn Diagrams in VennPainter

**Fig 4:**
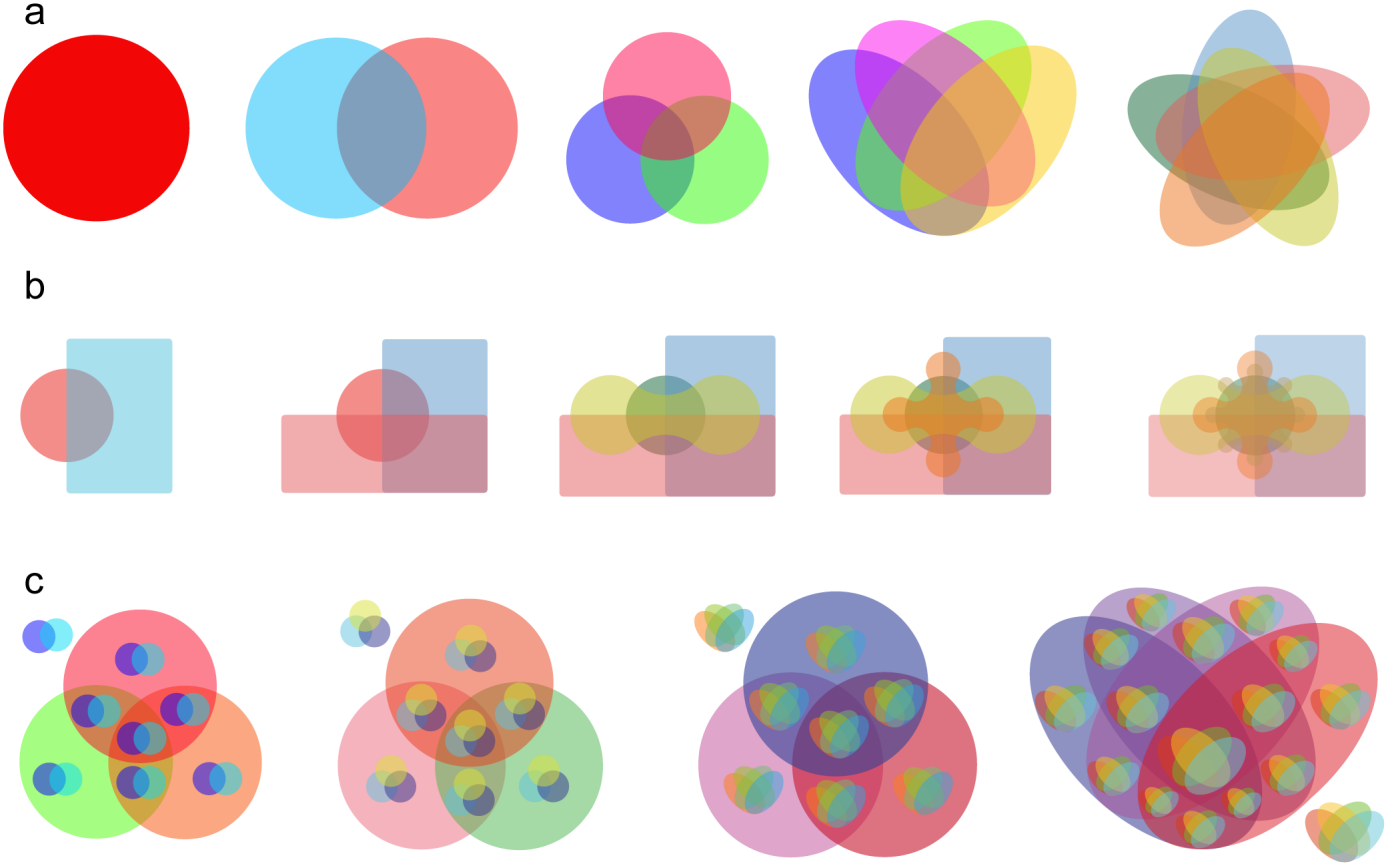
Venn diagrams and Nested Venn in VennPainter. **(a)** Classic Venn diagram depicting from one to five datasets. **(b)** Edwards’ Venn diagrams for from two to six datasets. **(c)** Nested Venn diagrams showing from five to eight variables. Nested Venn diagrams uses single-level Classic Venn diagrams to construct multi-level ones, which are easier to interpret than other forms of Venn diagrams when the datasets reaches more than six.

Users can select Classic Venn, Edwards’ Venn and Nested Venn diagrams [25] (Fig 4a–4c).

### Input and Output

**Fig 5:**
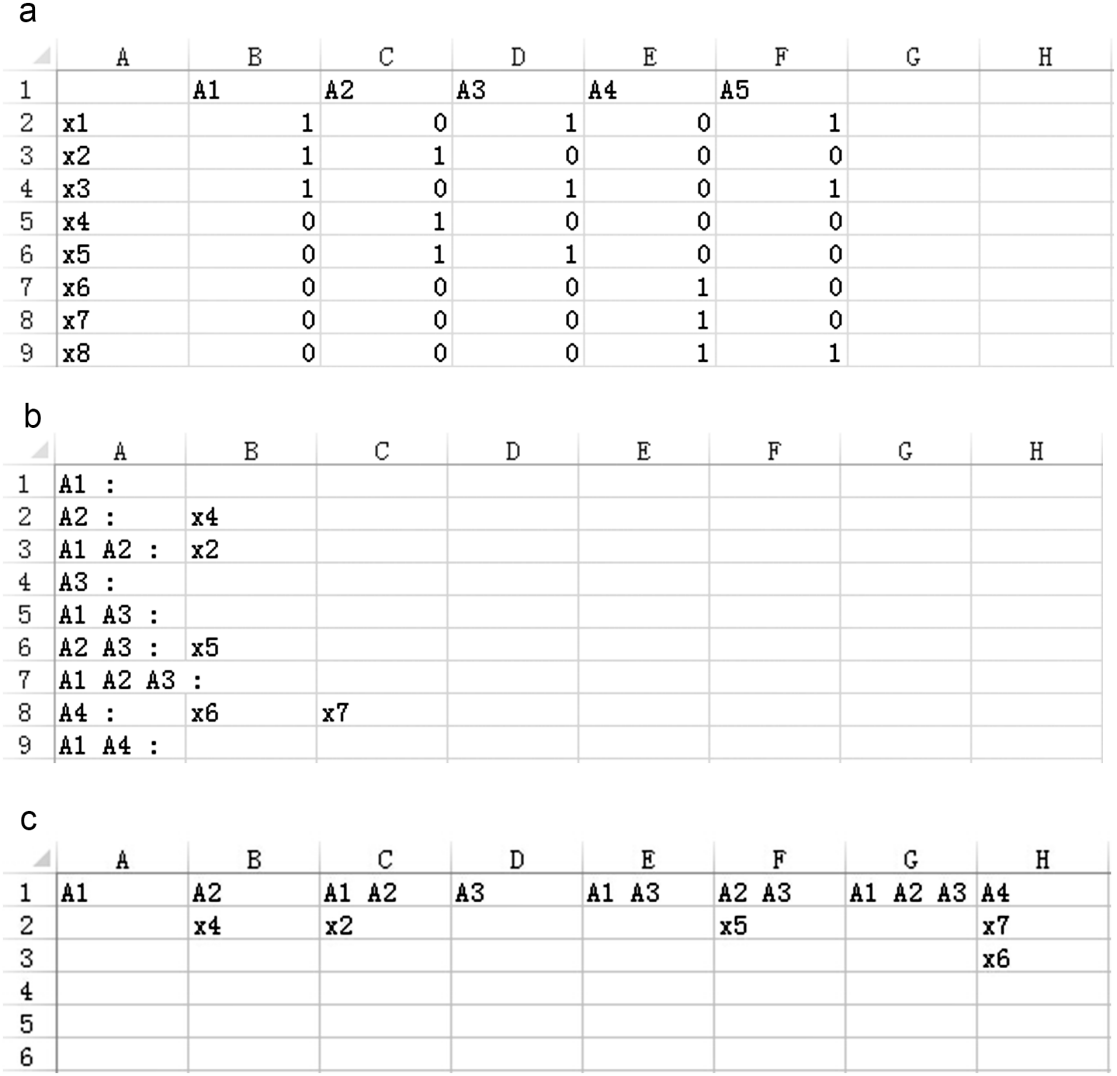
Output shared datasets. The Horizontal, Vertical and Matrix formats of output datasets. **(a)** Matrix format, first row contains all datasets and the first column contains all dataset elements; remaining columns denote if an element belongs to the dataset. Matrix used to construct a network. **(b)** Horizontal format, each line represents one intersection shared by datasets, which are listed before the colon. **(c)** Vertical format, identical to the Horizontal format with exchanged columns and rows.

VennPainter requires that each set be input as a text file. A white space character (space, tab, and newline) must separate every element in the set. After uploading all files, the program stores all elements in a hash table and classifies the elements. The algorithm obtains all statistics from a single read of the hash table. VennPainter can export integrated data as a text file (Fig 5) in Matrix, Vertical and Horizontal text-based formats. In the Matrix format, the first row contains all datasets and the first column contains all elements from the datasets. Other columns contain elements belonging to respective datasets. In the Vertical mode, each row indicates an intersection. For example, a six-set Venn diagram has 64 intersections and, thus, the text file contains 64 rows. Horizontal mode is identical to the vertical mode except for the exchange of columns and rows. Further, VennPainter can export single-shared datasets. Users can obtain a specific shared-dataset by clicking the number on the diagram and the ‘export’ button. Exported images are in the SVG format (Scalable Vector Graphics) [38,39], which can be read and modified easily by many graphic vector editors, such as Adobe Illustrator, Inkscape and CorelDRAW. The software provides tooltips when the mouse point over buttons or numbers in the diagram.

## Results and Discussion

### Example Application

To demonstrate the functions of VennPainter, we use it to depict shared gene sets in the goldfish x common carp hybrid system using eight annotated gene lists generated from RNA-seq data (S4 Fig) [37]. The Nested Venn diagram shows unique and shared relationships of eight sets by inlaying four unique-shared diagrams into the other four sets’ unique sharing diagram. The number in the center-most area (27,681) in the black rectangle shows the shared genes by all eight samples (S4 Fig). In a very intuitive manner, Nested Venn shows that each sample had more than 200 unique genes. It efficiently obtains candidate genes and facilitates downstream analyses of GO enrichment and KEGG annotation [37].

**Fig 6:**
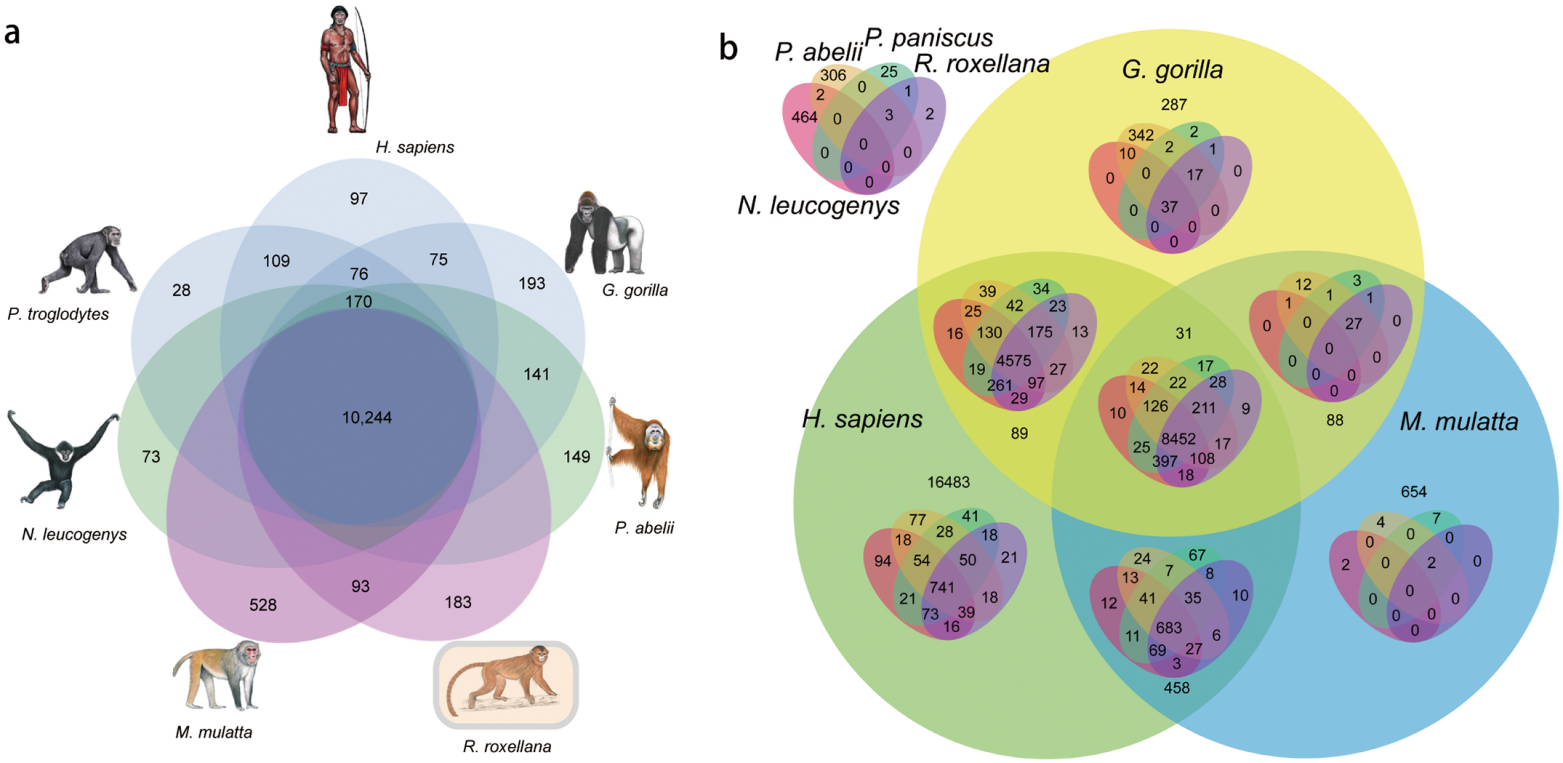
Comparisons of genes among seven primates. (a)The incomplete seven-sets Venn diagram generated by the method referenced in Zhou *et al*. (2014) [36]. It contains 38 unique or shared sets and many intersections are lost. It is not a general method of drawing a Venn diagram. (b) The full Nested Venn diagram depicting 127 regions was generated by VennPainter.

We evaluate the following seven primate gene-lists from GFF files (NCBI Genome database; S1 Table) using VennPainter: *Homo sapiens*, *Gorilla gorilla*, *Macaca mulatta*, *Nomascus leucogenys*, *Pongo abelii*, *Pan paniscus*, and *Rhinopithecus roxellana*. A comparison of our analyses with that of Zhou *et al*. 2014 [36] is informative. Analyses by the latter authors discovered 38 unique or shared sets, only 14 sets were marked with gene numbers, and 10,244 genes or gene families were shared by the seven primates (Fig 6a). In contrast, VennPainter depicts 127 intersections that a seven-set Venn diagrams should resolve, and these primates share 8,452 annotated genes (Fig 6b). Their Venn diagram did not depict all possible logical relationships among all the sets.

### Benchmark Test

To evaluate VennPainter’s relative performance, we use benchmarking data (Table 1). The benchmarking database contains four files of about 0.5MB each. Comparisons use an Intel core i5-5200U, 12GB memory, and Win 10 (64-bit). jVenn and Venny use Google Chrome 47.0.2526.111 m (64-bit). In comparison, jVenn, Venny and VennDiagram consume 3554 milliseconds (ms), 979 ms and 1078 ms, respectively, while VennPainter only costs 137 ms. Vennture crashes after 8.4*10^5^ms. Thus, VennPainter is more than seven times faster than other tested programs. The increased speed owes to VennPainter bring programmed in C++, while Venny and jVenn were programmed by JavaScript and VennDiagram by R.

**Table 1:** Comparison of BioVenn, Venny, jVenn, VennDiagrams, VennTure, and VennPainter.

	BioVenn	Venny	jVenn	VennDiagrams	VennTure	VennPainter
Application type	web application	web application	web application	R package	Standalone (Windows only)	Standalone (Cross-platform)
Fill-shape Color	Yes	Yes	Yes	Yes	No	Yes
Maximum sets	3	4	6	5	6	8 in graph, 31 in data
Image format	PNG and SVG	PNG	PNG	TIFF	EMF	SVG
Layouts	Classic	Classic	Classic and Edwards	Classic	Edwards	Nest Venn, Classic and Edwards
Interface	Graphical User Interface	Graphical User Interface	Graphical User Interface	Commend Line Interface	Graphical User Interface	Graphical User Interface
Performace with benchmark data	-^a^	979ms	3554ms	1078ms	8.4*10^5^ ms^b^	137ms

^a^: BioVenn is based on browser/server architecture. It is impossible to be estimated in local machine.

^b^: Time was estimated when VennTure ran out of memory (2GB). VennTure is a win32 program that cannot manage more than 2GB memory.

### Platforms and GUI

Several features make VennPainter more efficient at processing data than other available tools. VennPainter works with Windows, Linux and Mac operating systems (Table 1) and it has a concise GUI that eliminates the need for programming skills. The simple clicking on a number in any diagram promotes downstream analyses. Unlike other programs, VennPainter provides three diagrams including Classic Venn, Edwards’ Venn and Nested Venn diagrams for flexibility. Nested Venn is the default depiction when evaluating for more than six sets because regions have a more evenly distribution than Edwards’ Venn and are more orderly than classic Venn [34]. This approach makes it easy to fill in and visualize numbers. Nested Venn diagrams are particularly effective when considering more than six datasets, and VennPainter extends the capacity of processing up to eight datasets. So far, only VennPainter can achieve this comparison. Thus, VennPainter can applied to all shared data that need to be extract from dataset(s) for genomic and transcriptomic comparison.

## Supporting Information

S1 Fig Labeled Classic Venn diagram. This is an example of a labeled Classic Venn diagram with 5 sets.
(TIF)

S2 Fig Labeled Edwards’ Venn diagram. This is an example of labeled Edwards’ Venn diagram with 5 sets.
(TIF)

S3 Fig Labeled Nested Venn diagram. This is an example of labeled Nested Venn diagram with 5 sets.
(TIF)

S4 Fig Nested Venn with eight data sets. Example from the goldfish x common carp hybrid system with Nested Venn. The right smaller diagram in the green rectangle shows uniquely shared sets only among four datasets (f18, f22-1, f22-2, f22-3), while the larger left diagram includes all eight shared relationships by inlaying the right four into every intersection area showing another unique shared set among datasets for R♀, C♂, f1 and f2. For example, the number in the red rectangle, 53, which is over R♀ and f18, means that R♀ and f1 shared 53 items only. The Nested Venn diagram shows that each sample has more than 200 unique genes and all samples share 27,681 genes. Data sets are from Liu et al. (2016) [37].
(TIF)

S1 Table GFF file information. (PDF)
