# Supplementary figures and images for: VennPainter: A Tool for the Comparison and Identification of Candidate Genes Based on Venn Diagrams
Source: PLoS One. 2016 Apr 27;11(4):e0154315. doi: 10.1371/journal.pone.0154315 (PMC4847855; doi:10.1371/journal.pone.0154315)

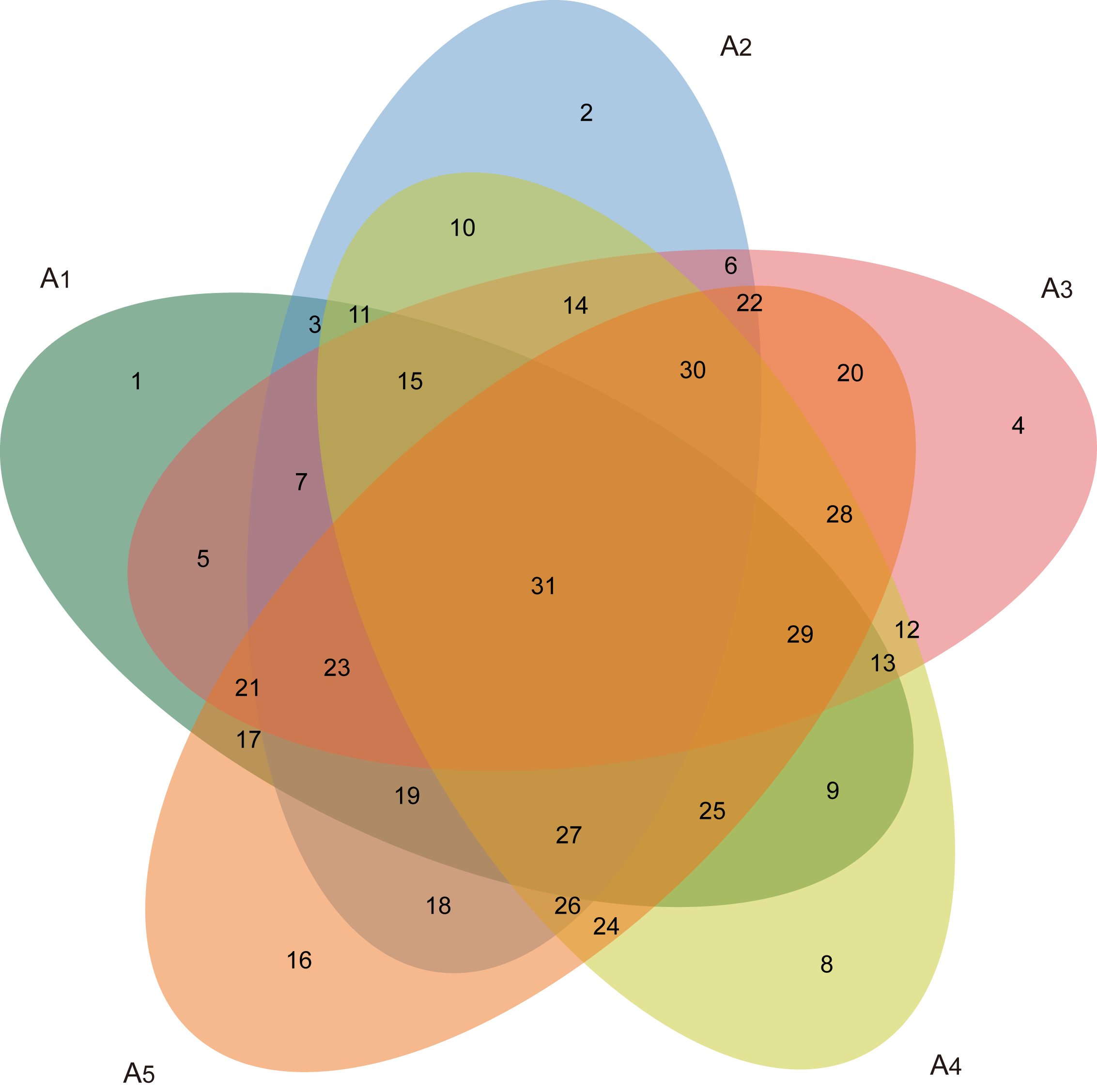

Supplement: S1 Fig — This is an example of a labeled Classic Venn diagram with 5 sets. (TIF) [file pone.0154315.s001.tif]

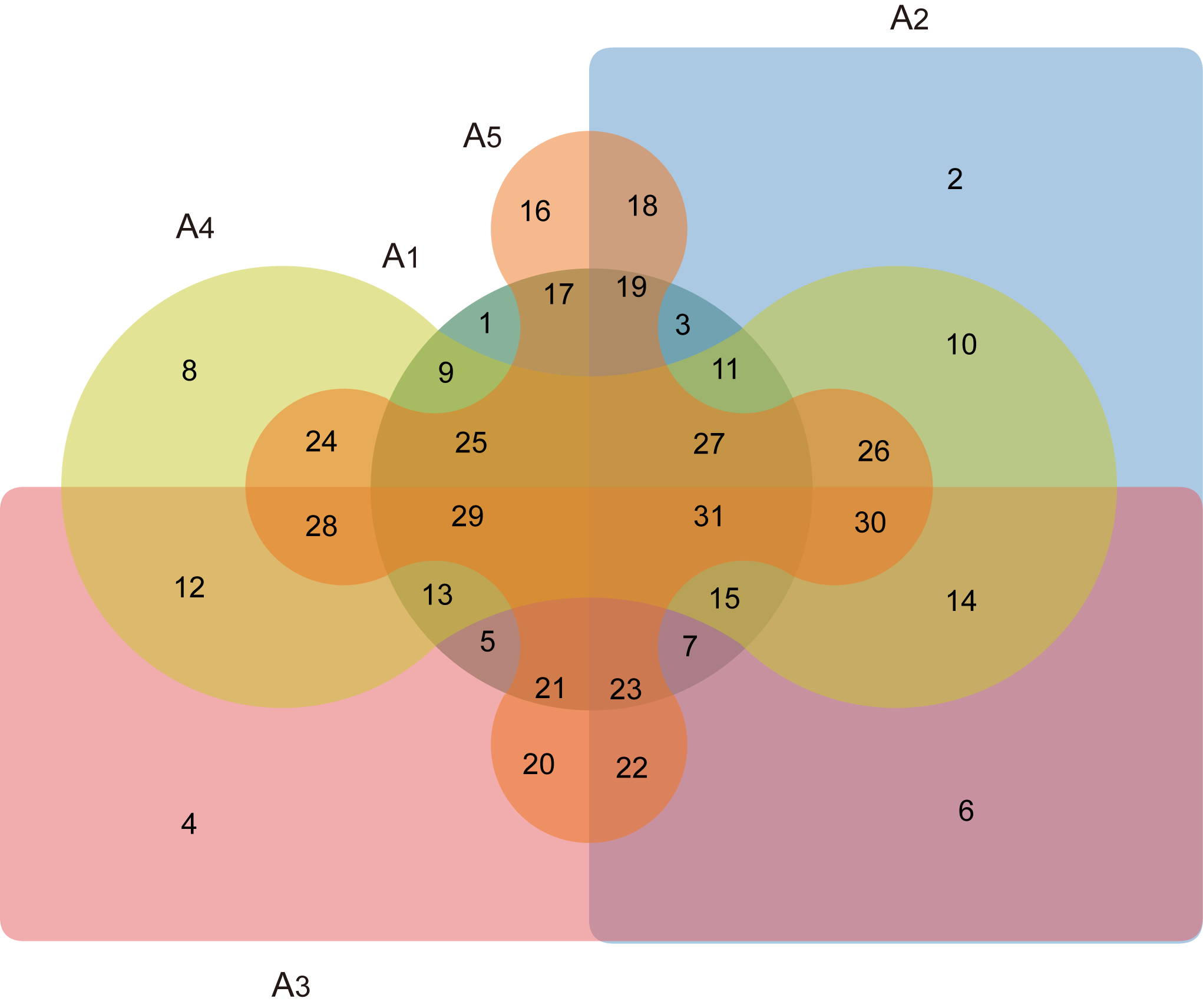

Supplement: S2 Fig — This is an example of labeled Edwards’ Venn diagram with 5 sets. (TIF) [file pone.0154315.s002.tif]

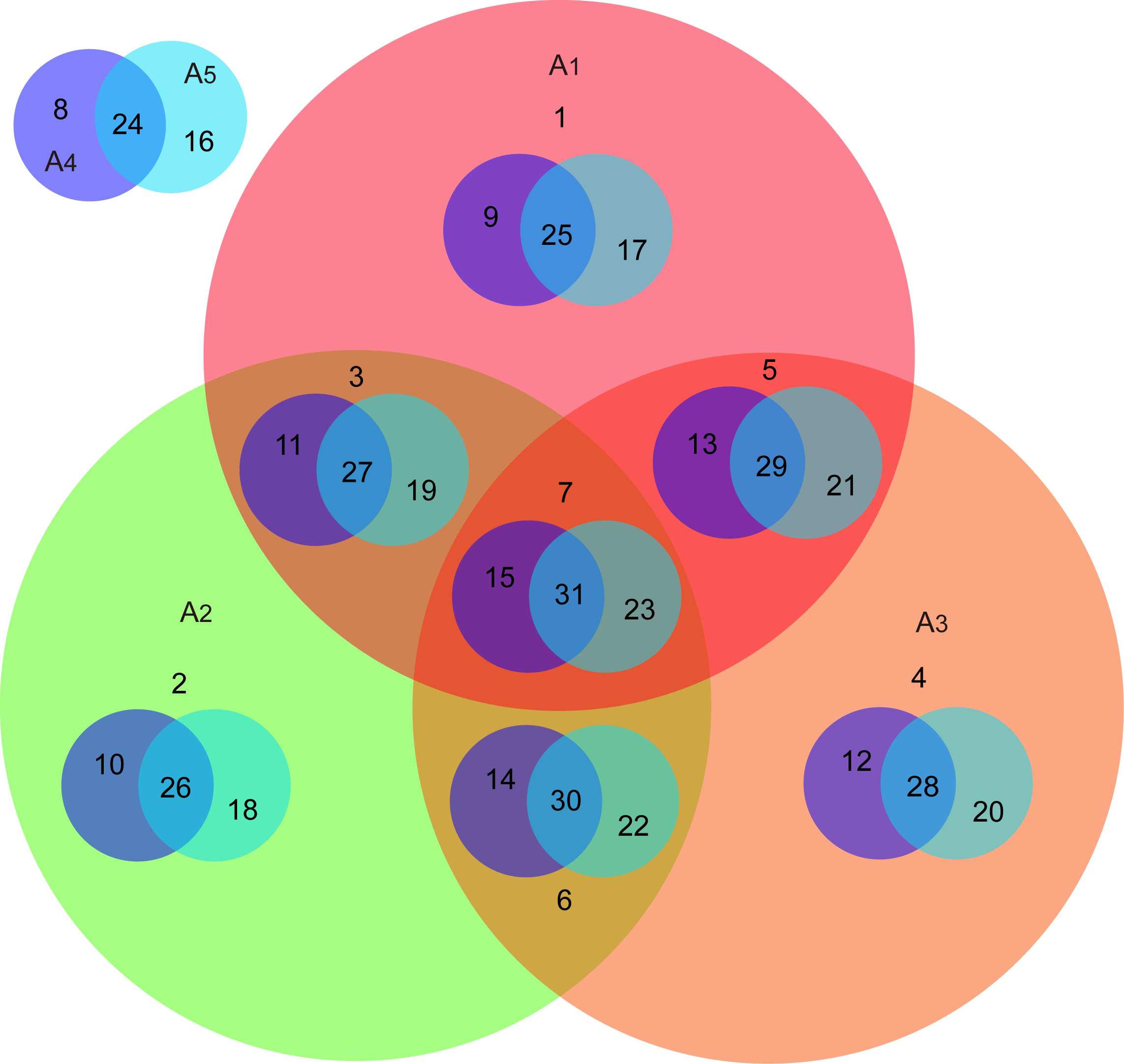

Supplement: S3 Fig — This is an example of labeled Nested Venn diagram with 5 sets. (TIF) [file pone.0154315.s003.tif]

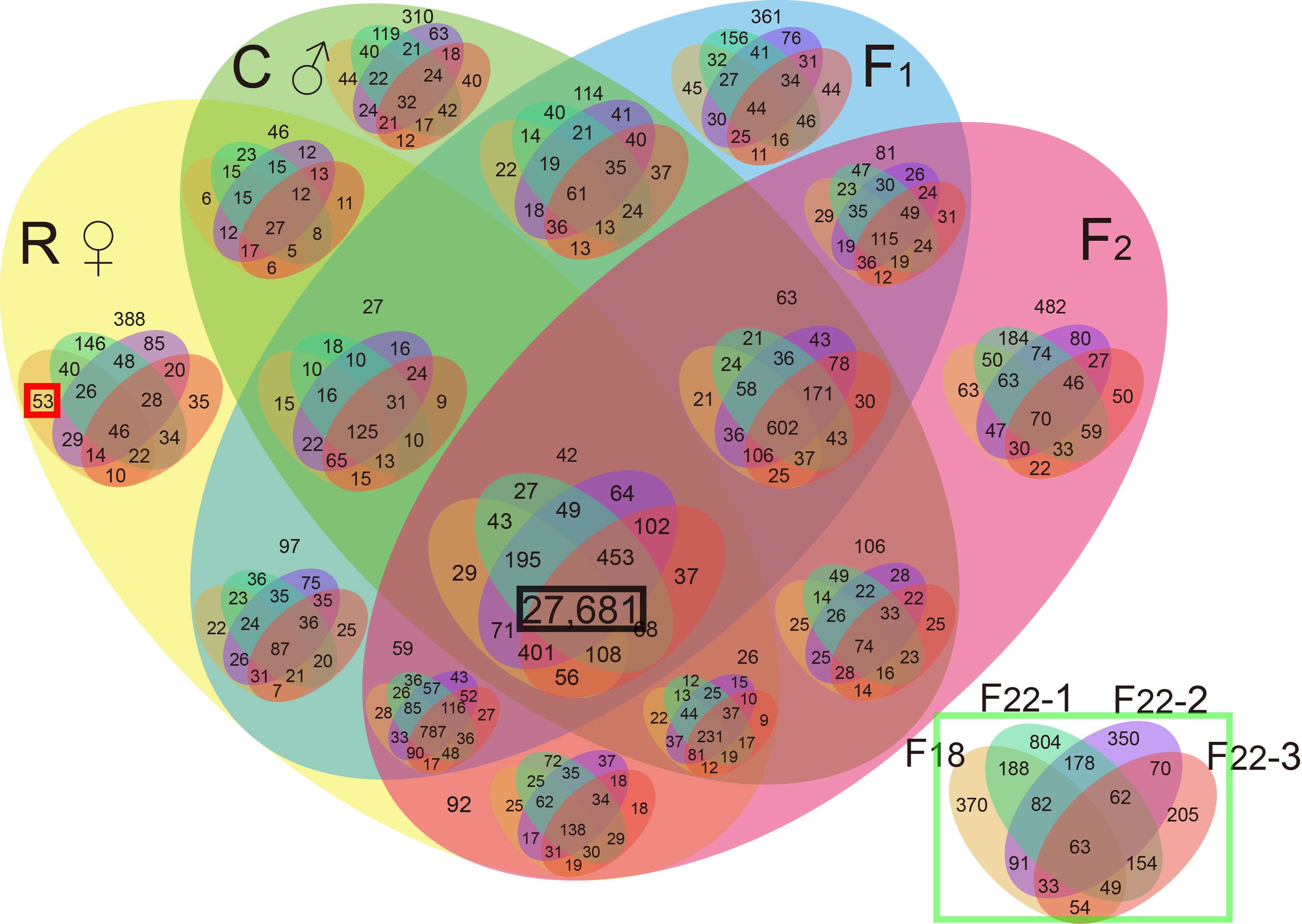

Supplement: S4 Fig — Example from the goldfish x common carp hybrid system with Nested Venn. The right smaller diagram in the green rectangle shows uniquely shared sets only among four datasets (f18, f22-1, f22-2, f22-3), while the larger left diagram includes all eight shared relationships by inlaying the right four into every intersection area showing another unique shared set among datasets for R♀, C♂, f1 and f2. For example, the number in the red rectangle, 53, which is over R♀ and f18, means that R♀ and f1 shared 53 items only. The Nested Venn diagram shows that each sample has more than 200 unique genes and all samples share 27,681 genes. Data sets are from Liu et al. (2016) [37]. (TIF) [file pone.0154315.s004.tif]
