# Supplementary material for: VennPainter: A Tool for the Comparison and Identification of Candidate Genes Based on Venn Diagrams
Source: PLoS One. 2016 Apr 27;11(4):e0154315. doi: 10.1371/journal.pone.0154315 (PMC4847855; doi:10.1371/journal.pone.0154315)
Supplement: S1 Table — (PDF) [file pone.0154315.s005.pdf]

Species	Assembly Version	download link
H. sapiens	GRCh38.p4	ftp://ftp.ncbi.nlm.nih.gov/genomes/all/GCF_000001405.30_GRCh38.p4/GCF_000001405.30_GRCh38.p4_genomic.gff.gz
G. gorilla	gorGor3.1	ftp://ftp.ncbi.nlm.nih.gov/genomes/all/GCF_000151905.1_gorGor3.1/GCF_000151905.1_gorGor3.1_genomic.gff.gz
M. mulatta	Mmul_051212	ftp://ftp.ncbi.nlm.nih.gov/genomes/all/GCF_000002255.3_Mmul_051212/GCF_000002255.3_Mmul_051212_genomic.gff.gz
N. leucogenys	Nleu_3.0	ftp://ftp.ncbi.nlm.nih.gov/genomes/all/GCF_000146795.2_Nleu_3.0/GCF_000146795.2_Nleu_3.0_genomic.gff.gz
P. abelii	P_pygmaeus_2.0.2	ftp://ftp.ncbi.nlm.nih.gov/genomes/all/GCF_000001545.4_P_pygmaeus_2.0.2/GCF_000001545.4_P_pygmaeus_2.0.2_genomic.gff.gz
P. paniscus	panpan1	ftp://ftp.ncbi.nlm.nih.gov/genomes/all/GCF_000258655.1_panpan1/GCF_000258655.1_panpan1_genomic.gff.gz
R. roxellana	Rrox_v1	ftp://ftp.ncbi.nlm.nih.gov/genomes/all/GCF_000769185.1_Rrox_v1/GCF_000769185.1_Rrox_v1_genomic.gff.gz
